# The Detection of Opioid Misuse and Heroin Use From Paramedic Response Documentation: Machine Learning for Improved Surveillance

**DOI:** 10.2196/15645

**Published:** 2020-01-03

**Authors:** José Tomás Prieto, Kenneth Scott, Dean McEwen, Laura J Podewils, Alia Al-Tayyib, James Robinson, David Edwards, Seth Foldy, Judith C Shlay, Arthur J Davidson

**Affiliations:** 1 Division of Scientific Education and Professional Development Centers for Disease Control and Prevention Atlanta, GA United States; 2 Denver Public Health Denver Health and Hospital Authority Denver, CO United States; 3 Department of Epidemiology Colorado School of Public Health Aurora, CO United States; 4 Denver Health Paramedics Denver Health and Hospital Authority Denver, CO United States; 5 Department of Family Medicine University of Colorado School of Medicine Aurora, CO United States; 6 Department of Biostatistics and Informatics Colorado School of Public Health Aurora, CO United States

**Keywords:** naloxone, emergency medical services, natural language processing, heroin, substance-related disorders, opioid crisis, artificial intelligence

## Abstract

**Background:**

Timely, precise, and localized surveillance of nonfatal events is needed to improve response and prevention of opioid-related problems in an evolving opioid crisis in the United States. Records of naloxone administration found in prehospital emergency medical services (EMS) data have helped estimate opioid overdose incidence, including nonhospital, field-treated cases. However, as naloxone is often used by EMS personnel in unconsciousness of unknown cause, attributing naloxone administration to opioid misuse and heroin use (OM) may misclassify events. Better methods are needed to identify OM.

**Objective:**

This study aimed to develop and test a natural language processing method that would improve identification of potential OM from paramedic documentation.

**Methods:**

First, we searched Denver Health paramedic trip reports from August 2017 to April 2018 for keywords naloxone, heroin, and both combined, and we reviewed narratives of identified reports to determine whether they constituted true cases of OM. Then, we used this human classification as reference standard and trained 4 machine learning models (random forest, k-nearest neighbors, support vector machines, and L1-regularized logistic regression). We selected the algorithm that produced the highest area under the receiver operating curve (AUC) for model assessment. Finally, we compared positive predictive value (PPV) of the highest performing machine learning algorithm with PPV of searches of keywords naloxone, heroin, and combination of both in the binary classification of OM in unseen September 2018 data.

**Results:**

In total, 54,359 trip reports were filed from August 2017 to April 2018. Approximately 1.09% (594/54,359) indicated naloxone administration. Among trip reports with reviewer agreement regarding OM in the narrative, 57.6% (292/516) were considered to include information revealing OM. Approximately 1.63% (884/54,359) of all trip reports mentioned heroin in the narrative. Among trip reports with reviewer agreement, 95.5% (784/821) were considered to include information revealing OM. Combined results accounted for 2.39% (1298/54,359) of trip reports. Among trip reports with reviewer agreement, 77.79% (907/1166) were considered to include information consistent with OM. The reference standard used to train and test machine learning models included details of 1166 trip reports. L1-regularized logistic regression was the highest performing algorithm (AUC=0.94; 95% CI 0.91-0.97) in identifying OM. Tested on 5983 unseen reports from September 2018, the keyword naloxone inaccurately identified and underestimated probable OM trip report cases (63 cases; PPV=0.68). The keyword heroin yielded more cases with improved performance (129 cases; PPV=0.99). Combined keyword and L1-regularized logistic regression classifier further improved performance (146 cases; PPV=0.99).

**Conclusions:**

A machine learning application enhanced the effectiveness of finding OM among documented paramedic field responses. This approach to refining OM surveillance may lead to improved first-responder and public health responses toward prevention of overdoses and other opioid-related problems in US communities.

## Introduction

### Background

The more than 47,000 opioid-involved overdose deaths in 2018 in the United States [1,2] insufficiently reflect the nonfatal burden associated with prescription opioid misuse and heroin use (OM) by an estimated 10.3 million people [3]. Timely, precise, and localized surveillance of nonfatal events is needed to define medical treatment trends related to OM and improve response and prevention of overdoses and other opioid-related problems.

Timely information sources about nonfatal opioid-related events include hospitals, emergency departments (EDs) [4], and prehospital emergency medical services (EMS). Paramedics routinely encounter patients with symptoms consistent with drug overdose and administer naloxone (an effective opioid antagonist) to reverse symptoms [5]. EMS data have helped estimate opioid overdose incidence, including nonhospital, field-treated cases [6-8]. Frequency of naloxone administration has positively correlated with opioid and heroin overdose-related ED visits [9] and fatal opioid overdose rates [10], suggesting that naloxone administration might be a relevant proxy to monitor need for interventions.

*Opioid misuse* and *heroin use* [11] refer to illicit use and nonmedical prescription opioid use for extended periods or for experience and feelings derived from the medication [12]. Naloxone, administered by paramedics to reverse opioid-induced respiratory depression [13,14], might serve as a potential OM sentinel, particularly when OM has resulted in an opioid overdose [5,9,10]. However, as naloxone is often used by EMS personnel in unconsciousness of unknown cause, attributing naloxone administration to opioid overdose and OM may misclassify events as opioid-related. A study of EMS-administered naloxone reported poor sensitivity and low positive predictive value (PPV) for opioid overdose [15].

### Objective

Better methods are needed to accurately identify opioid-related problems and trends of OM. To fill this gap, we sought to develop and test a natural language processing (NLP) method that would improve classification of OM among paramedic trip reports with documentation of naloxone administration or evidence of heroin use.

## Methods

### Setting

Denver Health’s (DH) [16] Paramedic Division is the main provider of EMS for the city and county of Denver. Their record system adheres to the National Emergency Medical Services Information System data standard version 3.4.0 [17]. We processed the following variables for each trip report: free-text narratives, primary impressions, alcohol or drug use note, and list of medications administered by paramedics. Table 1 summarizes the 3 study phases.

The Quality Improvement Committee of DH, which is endorsed by the Colorado Multiple Institutional Review Board at the University of Colorado, Denver, determined that this work did not constitute human subjects research.

**Table 1:** Summary of study phases to classify emergency medical services trip reports for potential opioid misuse, Denver, Colorado, 2017.

Phase	Purpose	Description of methods	Time frame
1	Assess performance of keyword search approaches	Searched trip reports for keywords (ie, “naloxone,” “heroin,” and both combined) and reviewed charts of identified reports to assess positive predictive value	August 2017 to April 2018
2	Train and test supervised machine learning classification	Guided machine learning models using previous phase’s chart review classification results and selected the highest performing algorithm in binary classification of opioid misuse and heroin use	August 2017 to April 2018
3	Validate performance measures across approaches	Compared the highest performing machine learning algorithm with the performance of searches of keywords “naloxone,” “heroin,” and combination of both	September 2018

### Phase 1: Assess Text String Search Approaches

Naloxone administrations have been previously used to flag potential OM resulting in opioid overdoses [5,9,10], and heroin use implies OM. To reduce the DH EMS dataset to a prescreened subset of all paramedic reports, we searched for presence of keywords *naloxone* (or *narcan*) among administered medications or *heroin* (or misspelled variations *herion* and *heroine*) in trip report narratives between August 1, 2017, and April 30, 2018. No opioid brand names (eg, Oxycontin or Tramadol) were used to identify opioid-related events. Trip reports that included the keywords were reviewed by 2 independent reviewers, both DH paramedics, to answer the question: “Is there narrative evidence (yes, no or unsure) of illicit opioid use or prescription OM (ie, use beyond clinical needs, for extended periods, or for experience and feelings derived from the medication)?” If unsure or when adverse events from opioids did not imply misuse, reviewers were to classify that report as negative. We hypothesized lower false-positive rates for the *heroin* vs *naloxone* methods because heroin use implies OM. To visualize trends, weekly potential OM paramedic trip report counts for each search approach were calculated. Pearson correlation coefficients (*r*) assessed correlation between weekly OM paramedic trip report counts by search approach and reviewer assessments.

### Phase 2: Train and Test Supervised Machine Learning Classification

Trip reports with *naloxone* among administered medications or *heroin* in narratives, plus reviewer agreement regarding OM in the narrative, served as our reference standard classification for training and validation of machine learning models; trip reports without reviewer agreement were omitted (examples in Multimedia Appendix 1). We removed the blank space between words in all variables, except in narratives, to create single-text entities (ie, *DenverHealth* instead of *Denver Health*). We stemmed words and removed stop words (eg, *the*, *a*, or *and*). To prevent overfitting, an 80% training set and 20% test set were created. Training corpus was converted into a document term matrix (terms as columns and documents as rows) that described the frequency of terms that occurred in narratives. To classify trip reports (OM evidence: yes or no), we used NLP machine learning models available from the caret Package [18] on R version 3.4.1 (ie, random forest, k-nearest neighbors, support vector machines, and L1-regularized logistic regression). Values of hyperparameters and parameters for each model were estimated using default configurations (ie, no hyperparameter tuning), which were optimized with 3 repeats of 5-fold cross-validation and then fit to the entire training set. We assessed performance of each model by calculating PPV, negative predictive value (NPV), true-positive rates (TPRs), true-negative rates (TNRs), and areas under the receiver operating characteristic curves (AUCs), and we selected the binary classification algorithm with the highest AUC for subsequent model assessment. Details can be found in authored R code in Multimedia Appendix 2.

### Phase 3: Validate Performance Measures Across Approaches

We searched for presence of the keywords *naloxone* (or *narcan*) among administered medications or *heroin* (or misspelled variations *herion* and *heroine*) in narratives of unseen September 2018 trip reports. Resulting trip reports were manually assessed following the same methodology as in phase 1. We then applied the machine learning classifier selected in phase 2 of the study to the reduced dataset of September 2018 trip reports. We hypothesized that machine learning models would decrease false-positive classifications of the combined *naloxone* and *heroin* search method because the algorithm would have learned and benefited from agreement in human assessments in phase 1. Reviewers’ assessment was used as a reference standard to calculate PPV for each approach.

## Results

### Phase 1 Findings

In total, 54,359 trip reports were filed, and 1.09% (594/54,359) indicated naloxone administration; reviewers agreed on assessment in 86.9% (516/594) of reports. Among trip reports with agreement, 56.6% (292/516) were considered to include information revealing OM.

Approximately 1.63% (884/54,359) of all trip reports mentioned *heroin* in the narrative. Reviewers agreed on potential OM assessment in 92.9% (821/884) of these. Among trip reports with agreement, almost all (784/821, 95.5%) were considered to include information revealing OM.

Combined results, where *naloxone* was administered by paramedics or *heroin* was mentioned in the narrative, accounted for 2.39% (1298/54,359) of trip reports. Reviewers agreed on potential OM assessment in trip reports in 89.83% (1166/1298) of these. Among trip reports with agreement, more than three-quarters (907/1166, 77.79%) included information consistent with OM.

**Figure 1:**
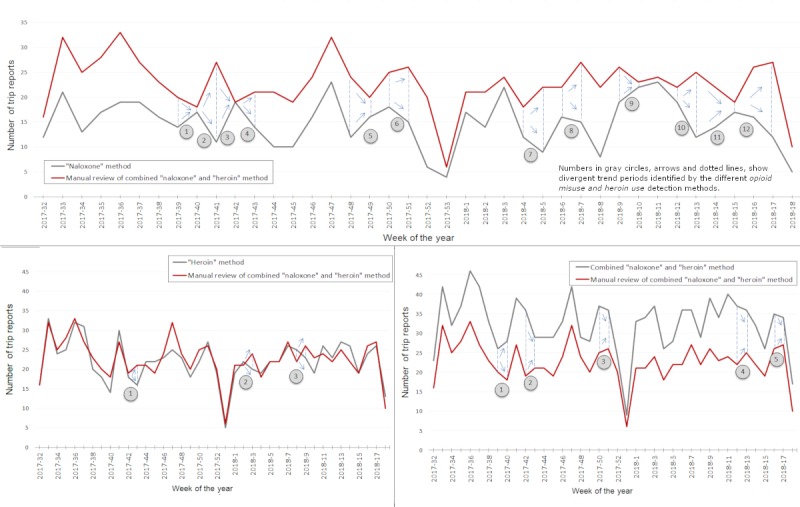
Weekly summary of paramedic trip reports trends for the documentation of administration of naloxone (top), heroin (bottom left), or both (bottom right), Denver Health, Denver, Colorado, August 1, 2017, to April 30, 2018.

Weekly counts of keywords mention varied by approach; Figure 1 is annotated to show periods of divergent trends between weekly sums of flagged reports and those affirmed by reviewer assessment. The *naloxone* approach was not consistent with reviewer assessment trends (*r*=0.60); the *heroin and* combined approaches were consistent with reviewer assessment trends (*r*=0.88 and *r*=0.90, respectively).

### Phase 2 Findings

The reference standard used to train and test machine learning models included details of 1166 *naloxone-* and *heroin*-flagged trip reports with positive OM reviewer assessment in phase 1. L1-regularized logistic regression was the highest performing algorithm (AUC=0.94; PPV=0.95; TPR=0.91; NPV=0.72; and TNR=0.84), followed by support vector machines (AUC=0.91; PPV=0.92; TPR=0.92; NPV=0.73; and TNR=0.73), random forest (AUC=0.91; PPV=0.91; TPR=0.95; NPV=0.79; and TNR=0.65), and k-nearest neighbors (AUC=0.81; PPV=0.79; TPR=1; NPV=0.1; and TNR=0.08). L1-regularized logistic regression yielded higher performance than the other algorithms; further statistical analyses, confusion matrices, and features that scored highest are presented in Multimedia Appendix 3.

### Phase 3 Findings

Among 5983 September 2018 trip reports, *naloxone* identified 63 events, and chart review revealed 20 false positives (PPV=0.68). Examples of false positives are presented in Multimedia Appendix 4. Keyword *heroin* identified 129 trip reports, and chart review revealed 1 false positive (PPV=0.99). Combined *naloxone* and *heroin* searches identified 171 trip reports with 20 false positives (PPV=0.88).

**Figure 2:**
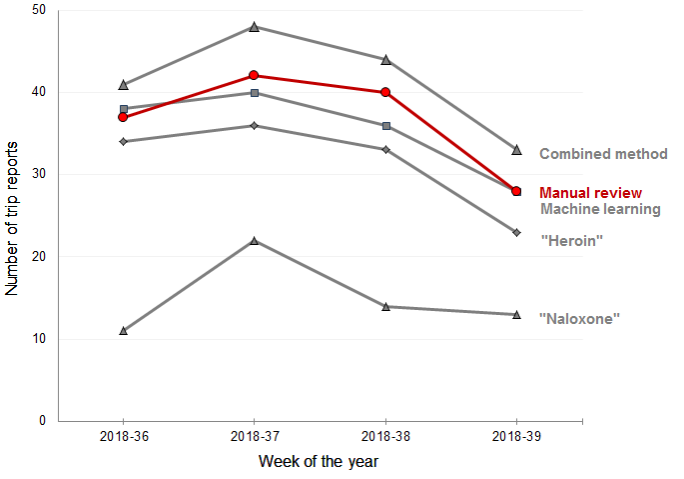
Trends in weekly number of potential opioid misuse events by detection method in paramedic trip reports, Denver, Colorado, September 2018.

L1-regularized logistic regression, the highest performing machine learning algorithm from phase 2, did not identify the one true negative of OM in reports flagged by *heroin* but identified 18 of the 20 true negatives of OM in reports flagged by naloxone administrations. The classifier identified 146 potential OM events from the 171 trip reports flagged by the combined text search with only 2 false positives. Results are summarized in Table 2. The machine learning classifier produced counts closer to those from reviewer assessment (Figure 2 shows counts for weeks 36 to 39 of 2018).

**Table 2:** Performance of natural language processing approaches to identify potential opioid misuse and heroin use in unseen September 2018 paramedic trip reports, Denver, Colorado.

Approach	Number of identified trip reports by approach (N)	Positive predictive value, n (%)	Correlation^a^ between weekly opioid misuse counts and chart review assessment
*Naloxone* search among administered medications	63	43 (68.3)	0.86
*Heroin* search in narratives	129	128 (99.2)	0.99
Combined search approach (*naloxone* or *heroin*)	171	151 (88.3)	1
Machine learning^b^ on combined search approach	146	144 (98.6)	0.99

^a^Pearson correlation coefficient.

^b^L1-regularized logistic regression.

## Discussion

### Principal Findings

This study sought to better understand documentation in paramedic trip reports as a tool to support more effective nonfatal OM surveillance. Accurate detection of potential OM events in survivors of EMS runs can reflect short-term trends in OM-related events at the community and national levels. These are potential leading indicators for assessing the nonfatal magnitude of the opioid crisis in an area.

Fluctuating supplies and introduction of powerful, illicitly manufactured opioids may rapidly change local morbidity and mortality patterns [19,20]. Availability of near real-time data of opioid-related problems from the field may guide prevention and intervention efforts of emergency responders, health care providers, and public health practitioners [4]. Our methods, similar to those used to identify opioid overdose risk [21], could be applied to enhance information accuracy of EMS data for state and local public health departments, an important goal in the Centers for Disease Control and Prevention (CDC) Emergency Response Cooperative Agreement [22].

Public health agencies in the United States are seeking data sources and data-driven indicators for early warning systems to identify medical consequences of misuse of prescription and illicit opioids [23]. Our study found that naloxone administrations inaccurately identified and underestimated opioid-related paramedic trip events in Denver. This result is compatible with recent findings that naloxone administration was a poor proxy for opioid overdose [15]. Our study also found that EMS-administered naloxone did not reflect trends (rise or fall) in OM-related EMS runs assessed by chart review. By itself, EMS naloxone administration was a poor stand-alone indicator and would benefit from additional information embedded in EMS records.

As a simple alternative, the keyword *heroin* increased over 2.5-fold (from 63 flagged by the current standard [ie, naloxone administrations] to 171) the number of records with potential OM. This strategy flagged OM reports accurately, with only 1 false positive. Combined *naloxone* and *heroin* NLP search increased sensitivity but with substantial false positives. To improve this, we applied a machine learning algorithm that produced both higher sensitivity and specificity. This same tactic, previously employed to identify alcohol misuse in clinical notes of electronic health records [24], could be extended to include more opioid-related terms such as prescription opioid names. New studies should try to assess the effects of including records flagged by keywords such as *heroin* or opioid brand names in model training, testing, and validation.

### Limitations

Two main limitations were present in this study. First, we used data from only 1 EMS system. Although DH paramedics adhere to a widely used data standard [17], implementation may vary between organizations. Second, calculation of the probability that cases not flagged by NLP methods were truly negative cases (NPV) was impossible as manual chart review of all trip reports would require human effort beyond our capacity.

## Appendix

Multimedia Appendix 1 Excerpts of narratives in paramedic trip reports without reviewer agreement.

Multimedia Appendix 2 R code used in phase 2.

Multimedia Appendix 3 Additional statistical analysis, confusion matrices, and feature scores by machine learning classifiers.

Multimedia Appendix 4 Excerpts of narratives of false positive results in phase 3.

## Abbreviations

AUC: area under the receiver operating curve
CDC: Centers for Disease Control and Prevention
DH: Denver Health
ED: emergency department
EMS: emergency medical services
NLP: natural language processing
NPV: negative predictive value
OM: opioid misuse
PPV: positive predictive value
TNR: true-negative rate
TPR: true-positive rate
