# Supplementary material for: The Detection of Opioid Misuse and Heroin Use From Paramedic Response Documentation: Machine Learning for Improved Surveillance
Source: J Med Internet Res. 2020 Jan 3;22(1):e15645. doi: 10.2196/15645 (PMC6969388; doi:10.2196/15645)
Supplement: Multimedia Appendix 1 [file jmir_v22i1e15645_app1.docx]

**Supplementary file 1:** Excerpts of narratives in paramedic trip reports without reviewer agreement

*Example 1*

“[…] Upon arrival patient found unconscious/unresponsive in wheelchair in front of Safeway. […]. Almost empty bottle of oxycodone found on patient, 2mg Narcan administered without improvement. […]”.

*Example 2*

“[…] Ambulance 2 called emergent for an unconscious party. […] DFD administered narcan prior to ambulance arrival. Patient moved to ambulance, patient color improved, remained unresponsive. Following and additional 2mg narcan patient skin condition improved, no change in mentation. […]
