# Supplementary material for: The Detection of Opioid Misuse and Heroin Use From Paramedic Response Documentation: Machine Learning for Improved Surveillance
Source: J Med Internet Res. 2020 Jan 3;22(1):e15645. doi: 10.2196/15645 (PMC6969388; doi:10.2196/15645)
Supplement: Multimedia Appendix 3 [file jmir_v22i1e15645_app3.docx]

**Supplementary file 3: Additional statistical analysis, confusion matrices, and feature scores by machine learning classifiers**

1. Additional statistical analysis:

We performed DeLong's test (Delong et al. 1988) for receiver operating characteristics (ROC) of the tested algorithms. We were interested in knowing if performance of the highest performing algorithm (L1-regularized logistic regression) was significantly higher than the performance of the other algorithms (at the 0.01 level). ROC of L1-regularized logistic regression (AUC = 0.94, 95% CI: 0.91-0.97) was not significantly different (*P* = 0.02) than ROC of support vector machines (AUC = 0.91, 95% CI: 0.86-0.95). It was not significantly different (*P* = 0.02) than ROC of random forest (AUC = 0.91, 95% CI: 0.86-0.96) either. However, it was significantly different (*P* < 0.001) than ROC of k-nearest neighbors (AUC = 0.91, 95% CI: 0.74-0.88).

*Reference:*

Elisabeth R. DeLong, David M. DeLong and Daniel L. Clarke-Pearson (1988) “Comparing the areas under two or more correlated receiver operating characteristic curves: a nonparametric approach”. Biometrics 44, 837--845.

2. Confusion matrices

*2.1. Confusion Matrix and Statistics for L1-regularized logistic regression*

Reference

Prediction No Yes

No 43 17

Yes 8 164

Accuracy : 0.8922

95% CI : (0.845, 0.929)

No Information Rate : 0.7802

P-Value [Acc > NIR] : 6.785e-06

Kappa : 0.7046

Mcnemar's Test P-Value : 0.1096

Sensitivity : 0.9061

Specificity : 0.8431

Pos Pred Value : 0.9535

Neg Pred Value : 0.7167

Prevalence : 0.7802

Detection Rate : 0.7069

Detection Prevalence : 0.7414

Balanced Accuracy : 0.8746

'Positive' Class : Yes

*2.2. Confusion Matrix and Statistics for k-nearest neighbors*

Reference

Prediction No Yes

No 4 0

Yes 47 181

Accuracy : 0.7974

95% CI : (0.7399, 0.8472)

No Information Rate : 0.7802

P-Value [Acc > NIR] : 0.2931

Kappa : 0.1172

Mcnemar's Test P-Value : 1.949e-11

Sensitivity : 1.00000

Specificity : 0.07843

Pos Pred Value : 0.79386

Neg Pred Value : 1.00000

Prevalence : 0.78017

Detection Rate : 0.78017

Detection Prevalence : 0.98276

Balanced Accuracy : 0.53922

'Positive' Class : Yes

*2.3. Confusion Matrix and Statistics for support vector machines*

Reference

Prediction No Yes

No 37 14

Yes 14 167

Accuracy : 0.8793

95% CI : (0.8303, 0.9183)

No Information Rate : 0.7802

P-Value [Acc > NIR] : 7.446e-05

Kappa : 0.6481

Mcnemar's Test P-Value : 1

Sensitivity : 0.9227

Specificity : 0.7255

Pos Pred Value : 0.9227

Neg Pred Value : 0.7255

Prevalence : 0.7802

Detection Rate : 0.7198

Detection Prevalence : 0.7802

Balanced Accuracy : 0.8241

'Positive' Class : Yes

*2.4. Confusion Matrix and Statistics for random forest*

Reference

Prediction No Yes

No 33 9

Yes 18 172

Accuracy : 0.8836

95% CI : (0.8352, 0.9219)

No Information Rate : 0.7802

P-Value [Acc > NIR] : 3.489e-05

Kappa : 0.6378

Mcnemar's Test P-Value : 0.1237

Sensitivity : 0.9503

Specificity : 0.6471

Pos Pred Value : 0.9053

Neg Pred Value : 0.7857

Prevalence : 0.7802

Detection Rate : 0.7414

Detection Prevalence : 0.8190

Balanced Accuracy : 0.7987

'Positive' Class : Yes

3. Twenty features that scored highest in the L1-regularized logistic regression:

| **Feature text*** | **Paramedics data field** | **Measure of importance**** |
| --- | --- | --- |
| chestbacklung | Narrative | 100.0 |
| Readi | Narrative | 70.0 |
| X1415 | Narrative | 67.9 |
| Alon | Narrative | 47.8 |
| Heroin | Narrative | 43.7 |
| Mucinex | Narrative | 39.6 |
| disorgan | Treatment list | 37.1 |
| cleara | Narrative | 34.9 |
| stolen | Narrative | 34.6 |
| buy | Narrative | 33.1 |
| opioid | Narrative | 31.0 |
| kit | Narrative | 30.4 |
| patientadmitstodrugus | Drug/Alcohol Usage | 30.1 |
| anybodi | Narrative | 29.7 |
| X4leadsinus | Narrative | 29.7 |
| nap | Narrative | 29.5 |
| unavail | Narrative | 25.9 |
| X1927 | Narrative | 25.0 |
| les | Narrative | 23.7 |
| epinephrine | Narrative | 22.1 |

* Features may be stemmed words or variables in paramedics data that were removed from whitespace or punctuation in phase 2 of the study. For example, the original text in the training set that produced the “chestbacklung” feature was “Chest/back=lungs clear and equal”, and “X1415” comes from the text "GCS 14-15" (“GCS” stands for “Glasgow Coma Scale”). The "X" at the beginning of the numeric features was automatically added by an R routine to be able to handle numbers as categorical text variables.

** We scaled the absolute value of the coefficients to have a maximum value of 100 (R Package caret, 2019). A higher number means that the feature text had a higher importance when predicting a positive or negative case of OM from free-text narratives.

Reference:

- R Package caret, 2019. https://cran.r-project.org/web/packages/caret/caret.pdf

Twenty features that scored highest in the random forest model:

| Feature text* | Paramedics data field | Measure of importance** |
| --- | --- | --- |
| heroin | Narrative | 100.0 |
| patientadmitstodrugus | Drug/Alcohol Usage | 40.9 |
| naloxon | Treatment list | 36.5 |
| intub | Narrative | 17.5 |
| use | Narrative | 15.3 |
| overdos | Primary impression | 14.3 |
| alter | Narrative | 13.9 |
| deni | Narrative | 13.7 |
| epinephrine | Narrative | 13.2 |
| tube | Narrative | 13.2 |
| narcan | Narrative | 12.9 |
| nonereport | Drug/Alcohol Usage | 12.6 |
| status | Narrative | 11.8 |
| unabl | Narrative | 10.8 |
| mental | Narrative | 9.4 |
| capnographi | Narrative | 9.0 |
| airway | Narrative | 8.8 |
| chang | Narrative | 7.9 |
| cpr | Narrative | 7.8 |
| epi | Narrative | 7.2 |

* Features may be stemmed words (i.e., “deni” may represent “denies” or “denied”, and “intub” may represent “intubation” or “intubed”) or variables that were removed from whitespace or punctuation in phase 2 of the study (i.e., “patientadmitstodrugus” comes from the original text “patient-admits-to-drug-use”).

** We scaled feature importance values to have a maximum value of 100 (R Package caret, 2019; R Package randomForest, 2018). A higher number means that the feature text had a higher importance when predicting a positive or negative case of OM from free-text narratives.

Reference:

- R Package caret, 2019. https://cran.r-project.org/web/packages/caret/caret.pdf

- R Package randomForest, 2018. https://cran.r-project.org/web/packages/randomForest/randomForest.pdf
