# Supplementary material for: The Detection of Opioid Misuse and Heroin Use From Paramedic Response Documentation: Machine Learning for Improved Surveillance
Source: J Med Internet Res. 2020 Jan 3;22(1):e15645. doi: 10.2196/15645 (PMC6969388; doi:10.2196/15645)
Supplement: Multimedia Appendix 4 [file jmir_v22i1e15645_app4.docx]

**Supplementary file 4:** Excerpts of narratives of false positive results in phase 3.

*Example 1*

“[…] called to bus stop for an unconscious party. U/A found a 50s male slouched over on the bus unconscious and responsive to very vigorous painful stimuli only. […] Medications: 2mg Narcan nasally with no response […]”

*Example 2*

“[…] Dispatched emergent to bus station at stated address on a report of down party. […] Marks consistent with IVDA to both arms. […] Narcan given as noted, with no resulting response. […]”
